# Epigallocatechingallate Inhibits Migration of Human Uveal Melanoma Cells via Downregulation of Matrix Metalloproteinase-2 Activity and ERK1/2 Pathway

**DOI:** 10.1155/2014/141582

**Published:** 2014-08-12

**Authors:** Chi-Wu Chang, Yi-Hsien Hsieh, Wei-En Yang, Shun-Fa Yang, Yueqin Chen, Dan-Ning Hu

**Affiliations:** ^1^Institute of Medicine, Chung Shan Medical University, Taichung 40201, Taiwan; ^2^Department of Ophthalmology, Chung Shan Medical University Hospital, Taichung 40201, Taiwan; ^3^Department of Biochemistry, School of Medicine, Chung Shan Medical University, Taichung 40201, Taiwan; ^4^Institute of Biochemistry and Biotechnology, College of Medicine, Chung Shan Medical University, Taichung 40201, Taiwan; ^5^Department of Clinical Laboratory, Chung Shan Medical University Hospital, Taichung 40201, Taiwan; ^6^Department of Medical Research, Chung Shan Medical University Hospital, Taichung 40201, Taiwan; ^7^Tissue Culture Center, New York Eye and Ear Infirmary of Mount Sinai, 310 E. 14th Street, New York, NY 10003, USA

## Abstract

The effects of epigallocatechingallate (EGCG) on the migration and expression of MMP-2 of uveal melanoma cells have not been reported. We studied this effect and relevant signaling pathways in a human uveal melanoma cell line (M17). MTT study found that EGCG did not affect the cell viability of M17 cells up to 100 *µ*M. Wound-healing assay showed that EGCG significantly reduced the migration of melanoma cells in a dose-dependent manner from 20 to 100 *µ*M. Gelatin zymography showed that secreted MMP-2 activity was dose-dependently inhibited by EGCG, whereas the MMP-2 expression at protein and mRNA levels was not affected as determined by western blot and RT-PCR analysis. EGCG significantly increased the expressions of MMP-2 endogenous inhibitors (TIMP-2 and RECK) in M17 cells. Western blot analysis of MAPK signal pathways showed that EGCG significantly decreased phosphorylated ERK1/2 levels, but not p38 and JNK levels, in melanoma cells. ERK1/2 inhibitors also reduced the migration and activity of MMP-2 in M17 cells. The present study suggested EGCG at nontoxic levels could inhibit migration of melanoma cells via downregulation of activities of secreted MMP-2 through the inhibition of the ERK1/2 phosphorylation. Therefore, EGCG may be a promising agent to be explored for the prevention of metastasis of uveal melanoma.

## 1. Introduction

Uveal melanoma is the most common primary malignant intraocular tumor in adults and eventually metastasizes to the liver in up to 50% of patients within 15 years after the initial diagnosis [1–3]. Various procedures have been reported for the treatment of metastatic uveal melanoma, but the response rate is very poor. Most uveal melanoma patients with liver metastasis die within 6 months and the median survival time after diagnosis of metastasis is only 3.6 months [2, 3]. Because of the poor prognosis of metastatic melanoma, new therapies for inhibiting cell migration, invasion, and metastasis are urgently required.

Tea is the most popular and polyphenol-rich beverage worldwide. Epigallocatechingallate (EGCG), the major polyphenol in green tea, has been considered to be the major active compound with chemopreventive properties [4]. Numerous previous studies showed that dietary phytochemicals or EGCG have many beneficial effects such as suppressing inflammatory processes [5], increasing antioxidant activity [6–10], inducing tumor cells apoptosis [11], and protecting cells from tumor development. Its cost effectiveness and natural abundance make it an attractive substance to investigate.

The tumorigenesis process and progression to metastasis is generally recognized as a multistep process in which the cellular and molecular mechanism changes [12]. Five major classes of proteases (serine, aspartic, cysteine, threonine, and metalloproteinases) are involved in cancer cell metastasis [12]. Matrix metalloproteinases (MMPs) are one of the major proteases in the degradation of extracellular matrix (ECM), especially MMP-2 and MMP-9. This process may affect the adhesive capacity between cancer cells, promote the migration of cancer cells, and lead to the metastasis [13]. Numerous studies have showed that the relative expression levels of MMPs seem to increase with tumorigenesis and have connected elevated MMP-2 and MMP-9 levels with an increased metastasis and invasion [14, 15]. High levels of MMP-2 expression have been demonstrated in many different cancers, including the liver [16], lung [17], colon [18], breast [19], prostate [20], skin [21], and ovary [22]. The activated MMP-2 expression was also correlated with cancer cell invasion and metastasis in various cancers [23–25]. In uveal melanoma, Cottam et al. [26] observed that most of cell lines secreted MMP-2 in vitro and the expression of MMP-2 was associated with a poor prognosis [27].

Multifunctional effects of EGCG in downregulation of MMP-2 by interfering with the activation, secretion, and regulation of the molecule have been demonstrated in many cancer cell types such as breast [28], oral [29], lung [30], and others. However, the effect of EGCG on the cell migration and MMP-2 secretion in human uveal melanoma cells has not been reported. The purpose of this study was to investigate the effect and mechanism of EGCG on cell migration in human uveal melanoma cells.

## 2. Materials and Methods

### 2.1. Cell Line and EGCG Treatment

M17 cell line, a human uveal melanoma cell line, was isolated from a primary choroidal melanoma patient and was established as an immortal cell line by us (DNH) in the Tissue Culture Center of the New York Eye and Ear Infirmary as previously reported [31]. M17 cells were maintained in Dulbecco's modified Eagle's medium (DMEM) culture medium supplemented with 10% fetal bovine serum (FBS), 100 U/mL of penicillin, and 100 *μ*g/mL streptomycin sulfate. Cells were incubated at 37°C in a CO_2_ regulated incubator in a humidified 95% air/5% CO_2_ atmosphere. Epigallocatechingallate (EGCG) and U0126 (ERK 1/2 inhibitor) were purchased from Sigma Chemical Co. (St. Louis, MO, USA). For EGCG treatments, stock solutions of EGCG were dissolved in dimethyl sulfoxide (DMSO) at concentrations of 50 mM, and cells were treated with EGCG at a final concentration between 0 and 100 *μ*M for 24 hours.

### 2.2. Cell Viability

MTT (3-(4,5-dimethylthiazol-2-yl)-2,5-diphenyl tetrazolium bromide) assay was performed to detect the cytotoxicity of EGCG. Cells were seeded in 24-well plate and treated with different concentration of EGCG at the range from 0 to 100 *μ*M for 24 hours. After 24 hours, the media were removed and the cells were washed with PBS, followed by the addition of MTT (0.5 mg/mL) to the culture medium for 4 hours at 37°C. Subsequently, the absorbance values were measured by a microplate photometer at 570 nm.

### 2.3. Wound-Healing Assay

An in vitro wound-healing assay was used to observe the migration of M17 cells after EGCG treatment. M17 cells were seeded into 6-well plate for 24 hours until they visibly reached confluence. Before EGCG treatment, a pipette tip was used to create a straight scratch on the plate to simulate a wound. The width of the remaining gap was captured using phase-contrast microscopy (×100) at 0, 24, 48, and 72 hours.

### 2.4. Western Blot Analysis

Cells were seeded into 6 cm dish. EGCG at different concentrations (0, 20, 40, 60, 80, and 100 *μ*M) was added. After 24 hours, total cell lysates were collected using lysis buffer containing a protease inhibitors cocktail and then lysed by sonication using an ultrasonic processor. Afterward, cells extracts were microcentrifuged at 13000 rpm for 20 min at 4°C and the supernatants were collected. The protein concentrations of total cell lysates were determined by Bradford assay. The cell extracts were separated by 10% polyacrylamide gel and transferred onto a nitrocellulose membrane. Subsequently, the membrane was incubated with 5% nonfat milk in TBST buffer for 1 h blocking and then the primary antibodies of MMP-2, TIMP-2, RECK, phosphor-ERK1/2, phosphor-JNK, phosphor-p38, total JNK, total p38, and total ERK1/2 were added. After incubating for overnight at 4°C, secondary antibodies with horseradish peroxidase were used for the indirect detection of specific primary antibody for 1 hour at room temperature. Finally, the protein expression was detected by chemiluminescence with an ECL detection kit. The relative photographic density was quantified by Multi Gauge V2.2 software.

### 2.5. Gelatin Zymography

The activities of MMP-2 in a condition medium and cell lysate were measured by gelatin zymography protease assays. Cells were plated at a density of 5 × 10^4^ cells/well in 24-well plates for 24 hours and then treated with indicated concentrations (0, 20, 40, 60, 80, and 100 *μ*M) of EGCG for another 24 hours. Otherwise, cells were pretreated with an indicated concentration of specific inhibitors, 10 *μ*M U0126 (ERK 1/2 inhibitor), for 60 min followed by incubation with or without 60 *μ*M EGCG for an additional 24 hours. For MMP-2 activity detection, conditioned medium and cell lysate from treated cells were prepared without boiling or reduction and subjected to electrophoresis with 8% SDS polyacrylamide gels containing 0.1% gelatin. After electrophoresis, the gels were washed with 2.5% Triton X-100 for 30 min to remove SDS and incubated in a reaction buffer (40 mM Tris-HCl, pH 8.0, 10 mM CaCl2, 0.02% NaN3) at 37°C for 16 hours. Finally, the gel was stained with Coomassie brilliant blue R-250 [29].

### 2.6. RNA Isolation, Semiquantity PCR, and Quantitative Real-Time PCR

Total RNA was isolated from 1 × 10^6^ M17 cells treated with and without EGCG using Trizol (Life Technologies, Grand Island, NY) according to the manufacturer's instructions. Total RNA (2 *μ*g) was reverse transcribed into cDNA by SuperScript III First-Strand Synthesis Supermix (Invitrogen, Carlsbad, CA). The PCR was performed in a reaction mixture containing 2 *μ*L cDNA, 0.2 mM dNTP mixture, 2 *μ*M of each of the primers, 1 U Taq DNA polymerase, and 1-fold concentration of Thermal Pol Buffer (New England BioLabs, MA, USA) by denaturation at 95°C for 5 min, followed by amplification of indicated cycles of 95°C for 30 sec, 62°C for 30 sec, and 72°C for 30 sec. The specific primer sequences for these genes are as follows: MMP-2: 5′-GGCCCTGTCACTCCTGAGAT-3′ (forward), 5′-GGCATCCAGGTTATCGGGG A-3′ (reverse) and TIMP-2: 5′-GGCGTTTTGCAATGCAGATGTAG-3′ (forward), 5′-CACAGGAGCCGTCACTTCTCTTG-3′ (reverse). Quantitative real-time PCR analysis was carried out using TaqMan one-step PCR Master Mix (Applied Biosystems). 100 ng of total cDNA was added per 25 *μ*L reactions with MMP-2 or GAPDH primers and TaqMan probes. The MMP-2 and GAPDH primers and probes were designed using commercial software (ABI PRISM Sequence Detection System; Applied Biosystems). Quantitative real-time PCR assays were carried out in triplicate on a StepOnePlus sequence detection system. The threshold was set above the nontemplate control background and within the linear phase of target gene amplification to calculate the cycle number at which the transcript was detected.

### 2.7. Statistical Analysis

For all of the measurements, analysis of variance followed by Scheffe posteriori comparison was used to assess the differences between control and cells treated with various concentration of EGCG. A difference at *P* < 0.05 was considered to be statistically significant and the experiments were repeated three times.

## 3. Results

### 3.1. Effect of EGCG on Cell Viability

**Figure 1:**
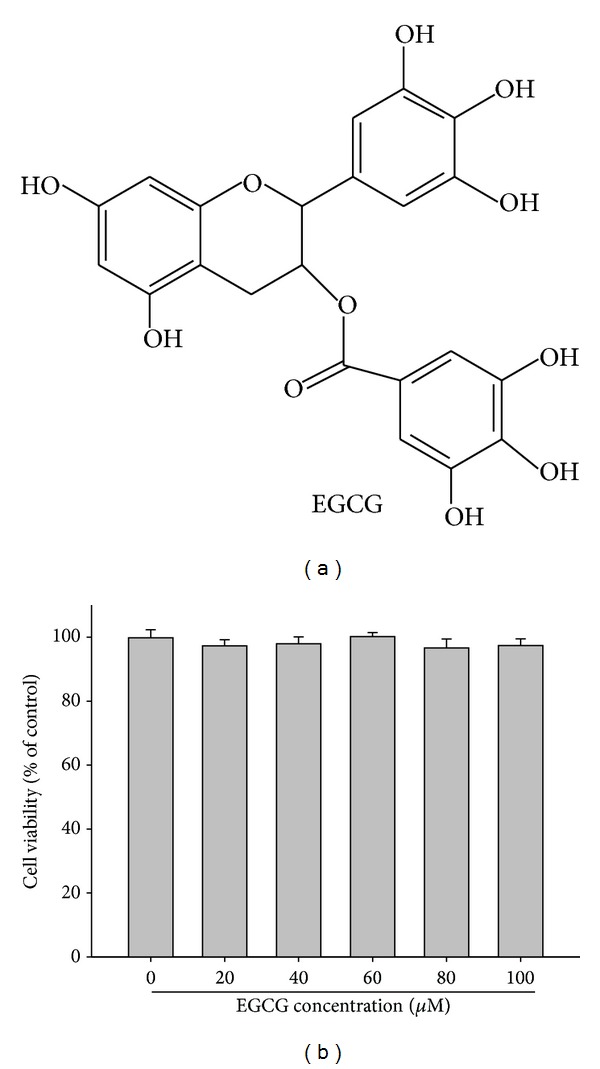
Effect of EGCG on cell viability. (a) Structure of EGCG. (b) Cell viability analysis of M17 cells cultured in presence of EGCG for 24 hours by MTT assay. M17 cells were treated with different concentrations of EGCG (0–100 *μ*M) for 24 hours. Data represent mean of 3 determinations per condition repeated 3 times. Results are shown as mean ± SD.

The chemical structure of EGCG is shown in Figure 1(a). Cytotoxic effects of EGCG on M17 cell was determined by MTT assay. As shown in Figure 1(b), after 24-hour treatment with various concentrations (0–100 *μ*M) of EGCG, the cell viability was not significantly affected. Therefore, a concentration range of 20–100 *μ*M of EGCG was chosen for subsequent experiments.

### 3.2. Effect of EGCG on Cell Migration

**Figure 2:**
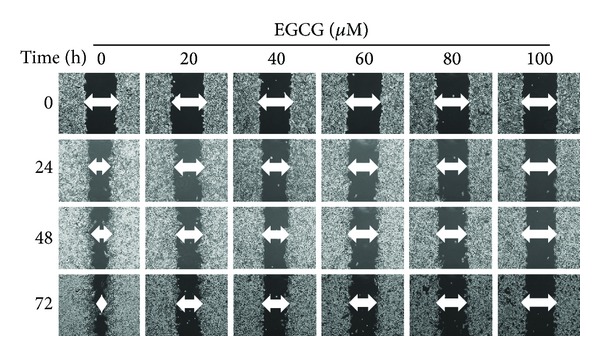
Effect of EGCG on cell migration in M17 cells. M17 cells were wounded and then treated with vehicle (DMSO) or EGCG (0, 20, 40, 60, 80, and 100 *μ*M) for 0 h, 24, 48, and 72 hours in 10% FBS-containing medium. At 0, 24, 48, and 72 hours, phase-contrast pictures of the wounds at three different locations were taken.

Wound-healing assay was used to investigate the migration of M17 cells cultured with or without EGCG. The results showed that EGCG significantly reduced the migration of M17 cells in a dose-dependent manner during 72 hours (Figure 2).

### 3.3. Effect of EGCG on the Expression and Secretion of MMP-2

**Figure 3:**
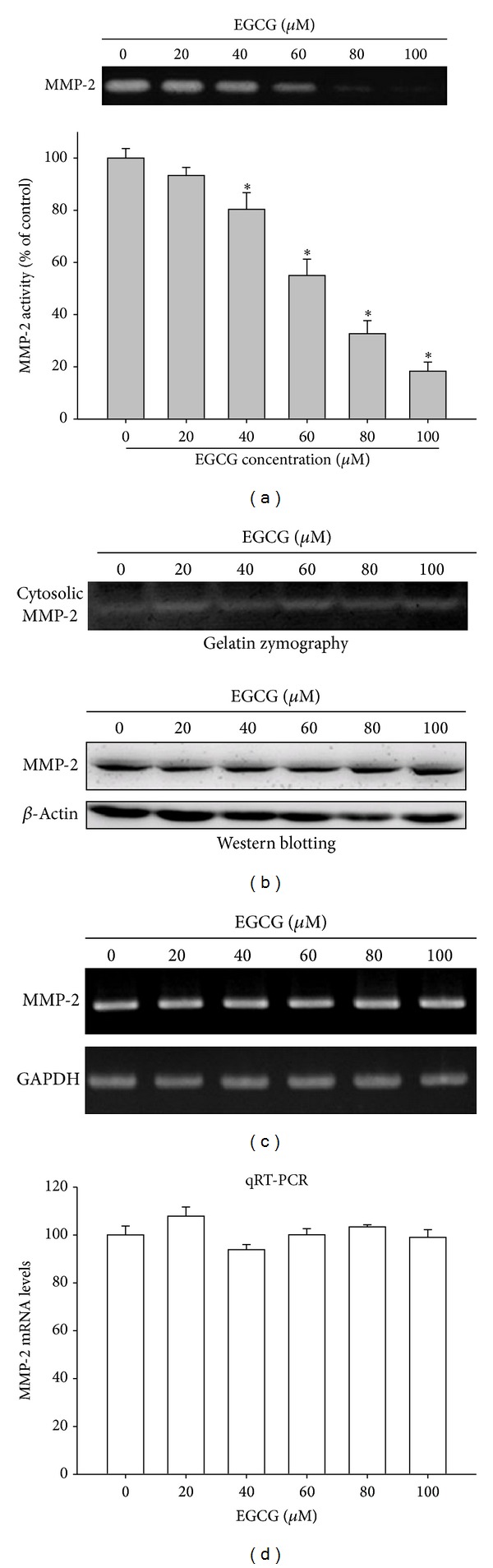
Effect of EGCG on the secretion, protein, and mRNA expression of MMP-2. M17 cells were treated with various concentrations (0, 20, 40, 60, 80, and 100 *μ*M) of EGCG for 24 hours. (a) The conditioned media were collected and the activity of MMP-2 was detected. (b) A gelatin zymography and western blot were performed for cell lysates. (c) Semiquantitative RT-PCR was performed to compare MMP-2 mRNA levels. (d) The mRNA levels of MMP-2 were quantified using a real-time PCR assay. The values represented the means ± SD of at least three independent experiments. *P < 0.05 as compared with the control.

Gelatin zymography was used to analyze the activity of secreted and cytosolic MMP-2 in M17 cells treated with and without EGCG.  Figure 3(a) shows that only secreted MMP-2 activity was significantly inhibited by EGCG in a dose-dependent manner. The protein and mRNA level of MMP-2 expression was indicated by western blot, semiquantity, and quantitative real-time PCR assay, respectively (Figures 3(b), 3(c), and 3(d)). The results showed that EGCG did not affect both protein and gene expressions of MMP-2. This suggested that EGCG inhibited activities of secreted MMP-2 but did not affect the expression of MMP-2.

### 3.4. Effect of EGCG on Endogenous Inhibitor Expression of MMP-2

**Figure 4:**
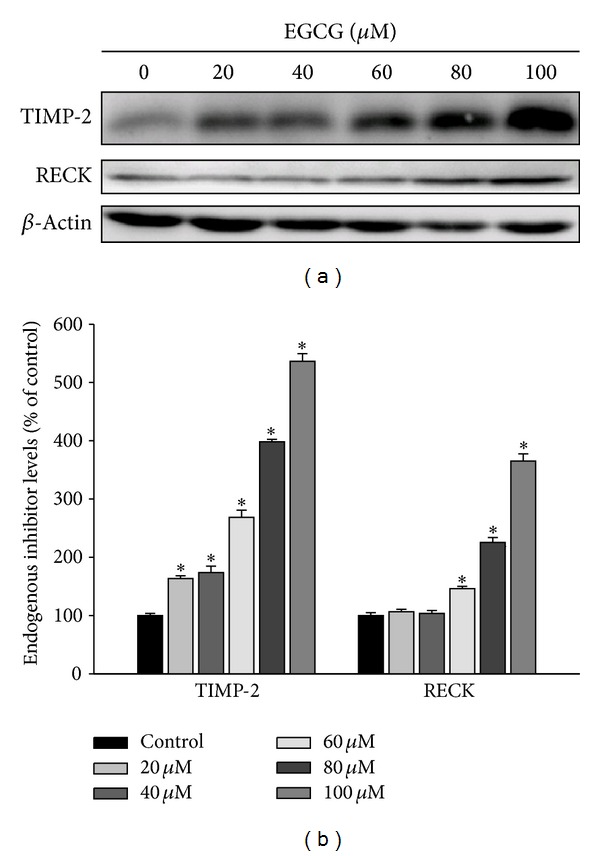
Effects of EGCG on the protein level of the endogenous inhibitor TIMP-2 and RECK. (a) M17 cells were treated with EGCG (0–100 *μ*M) for 24 hours and then subjected to western blotting to analyze the protein levels of TIMP-2 and RECK, respectively. (b) Quantitative results of TIMP-2 and RECK protein levels which were adjusted with *β*-actin protein level. The values represented the means ± SD of at least three independent experiments. *P < 0.05 as compared with the vehicle group.

From Figure 3, it was deduced that EGCG reduced the activities of secreted MMP-2. The physiological activities of MMP-2 are related to their specific endogenous inhibitors, tissue inhibitor of metalloproteinase- (TIMP-) 2, and reversion-inducing-cysteine-rich protein with kazal motifs (RECK). We analyzed the effect of EGCG on TIMP-2 and RECK expressions by western blot analysis. The results showed that EGCG significantly increased the expressions of TIMP-2 and RECK in M17 cells (Figure 4(b)). We further observed that EGCG substantially increased the TIMP-2 and RECK levels of M17 cells in a dose-dependent manner, with 5.36-fold and 3.65-fold increase after treating with 100 *μ*M of EGCG, respectively (Figure 4(b)).

### 3.5. Effect of EGCG on the Phosphorylation of ERK1/2 Pathway

**Figure 5:**
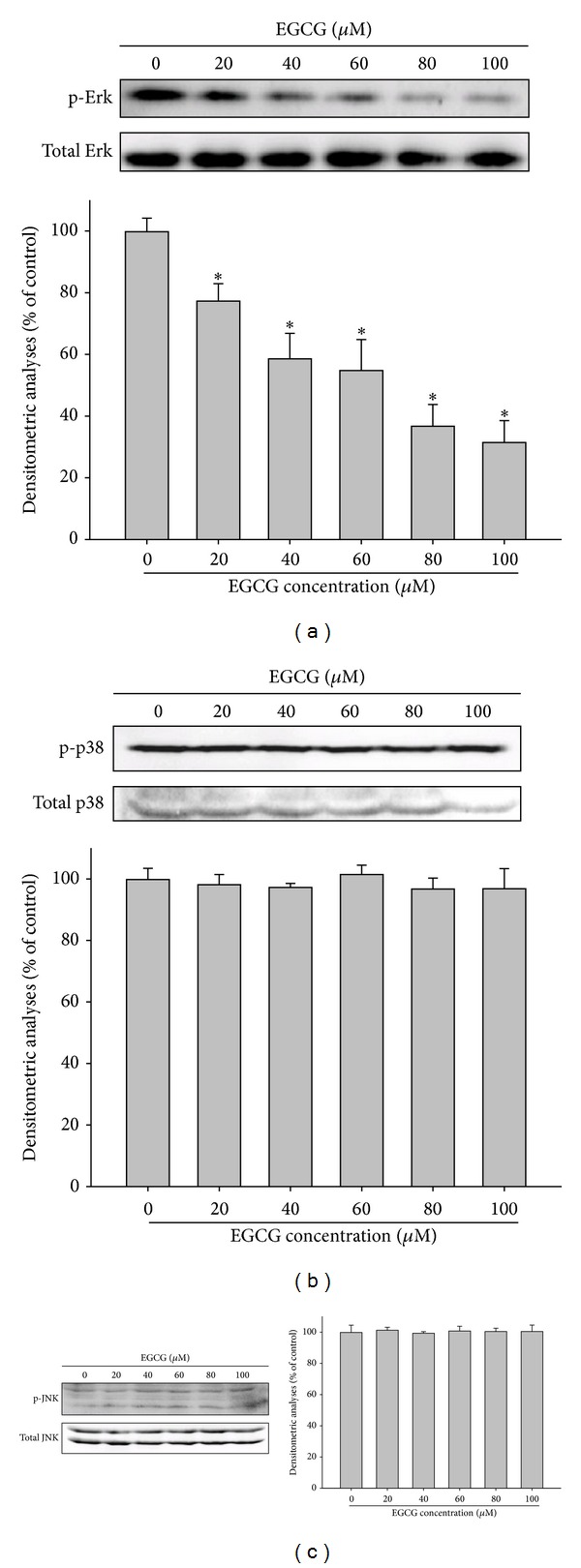
Effects of EGCG on the MAPK signaling pathways. M17 cells were treated with various doses of EGCG (0, 20, 40, 60, 80, and 100 *μ*M) for 24 hours and whole cell lysates prepared from these cells were used for western blot analysis with (a) anti-ERK, (b) anti-p38, and (c) anti-JNK (total and phosphorylated) antibodies as described in Materials and Methods section. The values represented the means ± SD of at least three independent experiments. *P < 0.05 as compared with the control.

As we have shown that the treatment of EGCG to M17 cells inhibited the cell migration and activities of secreted MMP-2, the underlying mechanisms were further investigated. As shown in Figure 5, the treatment of EGCG could significantly reduce the activation of phospho-ERK1/2 in a dose-dependent manner (Figure 5(a)). However, the phosphorylation of the JNK1/2 and p38 pathways remained unaffected (Figures 5(b) and 5(c)).

### 3.6. Inhibitory Effect of ERK1/2 Inhibitor on MMP-2 Activities and Cell Migration

**Figure 6:**
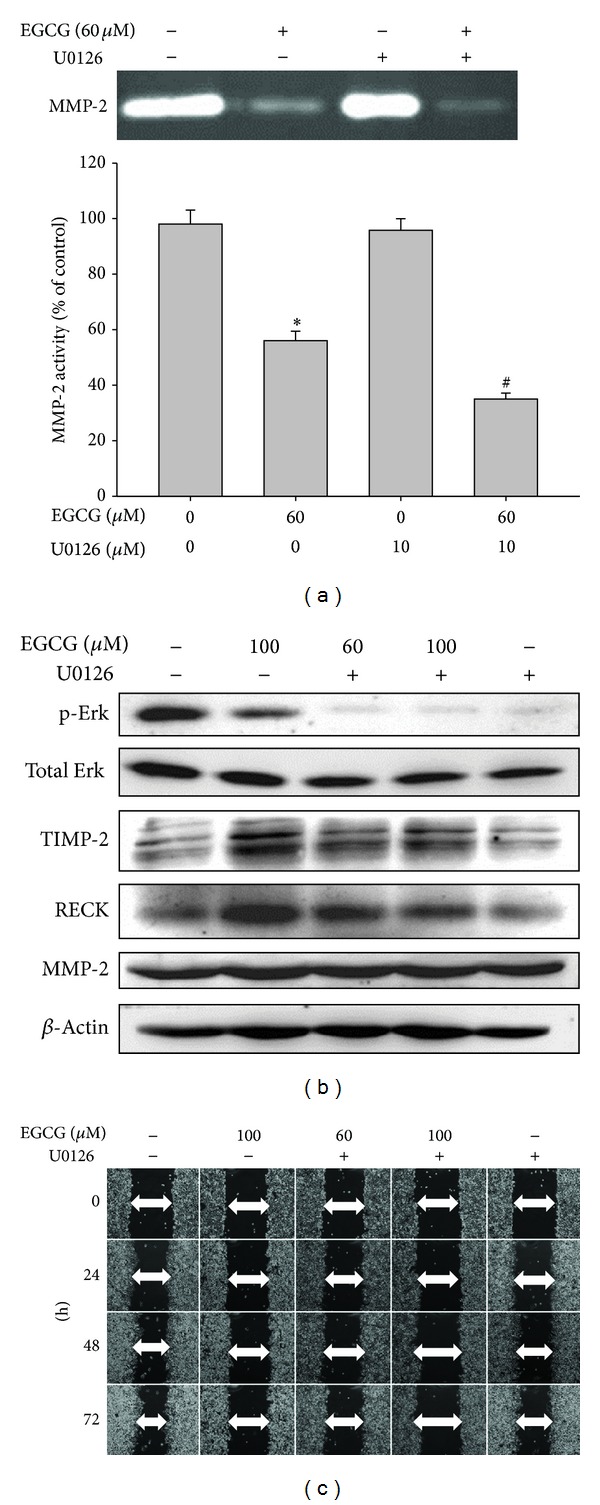
Effect of U0126 and EGCG on the expression of MMP-2, TIMP-2, and ERK1/2 pathway and cell migration. M17 cells were pretreated with U0126 (10 *μ*M) for 1 hour and then incubated in the presence or absence of EGCG (60 or 100 *μ*M) for 24 hours. (a) The culture media were used as subjects for analysis of MMP-2 activity. (b) The cell lysates were used as subjects for analysis of MMP-2, TIMP-2, and RECK protein levels. (c) Cells were used for wound-healing assay as described in Materials and Methods section. The values represented the means ± SD of at least three independent experiments. *P < 0.05 as compared with the control. ^#^ *P* < 0.05 as compared with the EGCG-treated only.

To further study whether the inhibition of MMP-2 activities by EGCG was mainly through an inhibition of ERK1/2 signaling pathway, we investigated the effects of specific inhibitor of ERK l/2 pathway (U0126) on M17 cells. Results showed that U0126 treatment led to the inhibition of MMP-2 activity, similar to the EGCG treatment (Figure 6(a)). In addition, a combined treatment of ERK1/2 inhibitor with EGCG could further decrease the MMP-2 activity (Figure 6(a)) and increase the expression of TIMP-2 and RECK protein but not affect the MMP-2 expression (Figure 6(b)). Furthermore, a similar result for an inhibition on the cell migration of M17 cells by a sole treatment of ERK1/2 inhibitor and combination treatment with ERK1/2 inhibitor and EGCG was also observed (Figure 6(c)). This suggested that the inhibition of ERK l/2 signaling pathways could result in the inhibition of activities of secreted MMP-2 as well as the cell migration of melanoma cells.

## 4. Discussion

M17 uveal melanoma cell line was isolated and cultured from a primary choroidal melanoma patient, has been cultured in vitro for more than 20 years, and has been divided more than 200 times, indicating that this is an immortal cell line. Cells grew actively, with a doubling time of 24–48 hours, and are tumorigenic in immune nude mice. This cell line has been used widely in several melanoma research centers for the study of melanogenesis, role of microRNA in the pathogenesis of uveal melanoma, and various pharmacological and toxicological studies [32–39]. Therefore, M17 cell line was selected for use in the present study. The effects of EGCG on the growth, migration, and invasion of cutaneous melanoma cells have been reported [40–42]. However, the effect of EGCG on the migration and expression of MMPs in uveal melanoma cells has not been reported.

Cutaneous melanomas are biologically different from uveal melanomas in many respects. Most cutaneous melanomas occur in area exposed to sun radiation, whereas most of the uveal melanomas occur in the posterior segment of the eye and are not exposed to sun radiation. UV radiation increases the incidence of cutaneous melanoma but not uveal melanoma [43, 44]. Gene mutations in cutaneous melanoma (BRAF, N-Ras, etc.) were entirely different from those in uveal melanoma (GNAQ, GNA11, etc.) [45]. Furthermore, the karyotypes of cutaneous melanomas are also different from those of uveal melanomas [46]. Therefore, cutaneous melanoma and uveal melanoma should be considered as two different and independent disease entities. Independent studies are required for each type of melanoma to develop relevant novel treatments.

The present study revealed that EGCG inhibited the migration and decreased the MMP-2 activity of uveal melanoma cells. To our knowledge, this study provides the first demonstration that EGCG is capable of inhibiting invasive behaviors and MMP-2 activities in uveal melanoma cells.

Previous studies have well established the role of the mitogen-activated protein kinase (MAPK) pathway in regulating MMP-2 expression [47]. Lin et al. indicated that kaempferol reduces MMP-2 expression by downregulating ERK1/2 signaling pathways in oral cancer cells [47]. Our previous study also showed that silibinin inhibits the invasion of oral cancer cells by suppressing the activation of ERK1/2 and MMP-2 expression [48]. Furthermore, in the study by Sen et al., EGCG downregulated MMP-2 in human breast cancer cell line via Erk 1/2 signal pathway [28]. In another study, JNK 1/2 pathway modulated MMP-2 production by EGCG treatment in lung cancer cells [30]. However, the present study showed that EGCG only inhibited ERK phosphorylation and no significant effects were detected on the JNK and p38 signaling pathways. The involvement of the MAPK pathway in the modulation of MMP-2 activities was demonstrated by treating uveal melanoma cells by ERK inhibitor, which showed that ERK inhibitor could lead to an inhibition of MMP-2 secretion and cell invasion of uveal melanoma cells.

In conclusion, our study results suggested that one of the antimetastatic effects of EGCG on uveal melanoma cells was the downregulation of activities of secreted MMP-2 through the inhibition of ERK1/2 phosphorylation. Overall, these data suggest that EGCG may be a promising agent to be explored for the prevention of metastasis of uveal melanoma.

## References

[1] Hu D, Yu G, McCormick SA, Schneider S, Finger PT (2005). Population-based incidence of uveal melanoma in various races and ethnic groups. *The American Journal of Ophthalmology*.

[2] Kujala E, Mäkitie T, Kivelä T (2003). Very long-term prognosis of patients with malignant uveal melanoma. *Investigative Ophthalmology and Visual Science*.

[3] Augsburger JJ, Corrêa ZM, Shaikh AH (2009). Effectiveness of treatments for metastatic uveal melanoma. *American Journal of Ophthalmology*.

[4] Singh BN, Shankar S, Srivastava RK (2011). Green tea catechin, epigallocatechin-3-gallate (EGCG): mechanisms, perspectives and clinical applications. *Biochemical Pharmacology*.

[5] Liu Q, Qian Y, Chen F (2014). EGCG attenuates pro-inflammatory cytokines and chemokines production in LPS-stimulated L02 hepatocyte. *Acta Biochimica et Biophysica Sinica*.

[6] Zhong Y, Shahidi F (2011). Lipophilized epigallocatechin gallate (EGCG) derivatives as novel antioxidants. *Journal of Agricultural and Food Chemistry*.

[7] Singh BN, Singh HB, Singh A, Naqvi AH, Singh BR (2014). Dietary phytochemicals alter epigenetic events and signaling pathways for inhibition of metastasis cascade. *Cancer and Metastasis Reviews*.

[8] Singh BN, Singh BR, Singh RL (2009). Oxidative DNA damage protective activity, antioxidant and anti-quorum sensing potentials of Moringa oleifera. *Food and Chemical Toxicology*.

[9] Singh HB, Singh BN, Singh SP, Nautiyal CS (2010). Solid-state cultivation of *Trichoderma harzianum* NBRI-1055 for modulating natural antioxidants in soybean seed matrix. *Bioresource Technology*.

[10] Prakash D, Upadhyay G, Singh BN, Singh HB (2007). Antioxidant and free radical-scavenging activities of seeds and agri-wastes of some varieties of soybean (Glycine max). *Food Chemistry*.

[11] An Z, Qi Y, Huang D (2014). EGCG inhibits Cd(2+)-induced apoptosis through scavenging ROS rather than chelating Cd(2+) in HL-7702 cells. *Toxicology Mechanisms and Methods*.

[12] Chambers AF, Matrisian LM (1997). Changing views of the role of matrix metalloproteinases in metastasis. *Journal of the National Cancer Institute*.

[13] Duffy MJ, McGowan PM, Gallagher WM (2008). Cancer invasion and metastasis: changing views. *The Journal of Pathology*.

[14] Schmalfeldt B, Prechtel D, Härting K (2001). Increased expression of matrix metalloproteinases (MMP)-2, MMP-9, and the urokinase-type plasminogen activator is associated with progression from benign to advanced ovarian cancer. *Clinical Cancer Research*.

[15] Mendes O, Kim H, Stoica G (2005). Expression of MMP2, MMP9 and MMP3 in breast cancer brain metastasis in a rat model. *Clinical and Experimental Metastasis*.

[16] Ogasawara S, Yano H, Momosaki S (2005). Expression of matrix metalloproteinases (MMPs) in cultured hepatocellular carcinoma (HCC) cells and surgically resected HCC tissues. *Oncology Reports*.

[17] Guo C, Wang S, Deng C, Zhang D, Wang F, Jin X (2007). Relationship between matrix metalloproteinase 2 and lung cancer progression. *Molecular Diagnosis and Therapy*.

[18] Papadopoulou S, Scorilas A, Arnogianaki N (2001). Expression of gelatinase-A (MMP-2) in human colon cancer and normal colon mucosa. *Tumor Biology*.

[19] Pellikainen JM, Ropponen KM, Kataja VV, Kellokoski JK, Eskelinen MJ, Kosma V (2004). Expression of matrix metalloproteinase (MMP)-2 and MMP-9 in breast cancer with a special reference to activator protein-2, HER2, and prognosis. *Clinical Cancer Research*.

[20] Trudel D, Fradet Y, Meyer F, Harel F, Têtu B (2003). Significance of MMP-2 expression in prostate cancer: an immunohistochemical study. *Cancer Research*.

[21] O'Grady A, Dunne C, O'Kelly P, Murphy GM, Leader M, Kay E (2007). Differential expression of matrix metalloproteinase (MMP)-2, MMP-9 and tissue inhibitor of metalloproteinase (TIMP)-1 and TIMP-2 in non-melanoma skin cancer: implications for tumour progression. *Histopathology*.

[22] Yu AE, Hewitt RE, Kleiner DE, Stetler-Stevenson WG (1996). Molecular regulation of cellular invasion-role off gelatinase A and TIMP-2. *Biochemistry and Cell Biology*.

[23] Hofmann UB, Westphal JR, Zendman AJW (2000). Expression and activation of matrix metalloproteinase-2 (MMP-2) and its co-localization with membrane-type 1 matrix metalloproteinase (MT1-MMP) correlate with melanoma progression. *The Journal of Pathology*.

[24] Stearns M, Steams ME (1996). Evidence for increased activated metalloproteinase 2 (MMP-2a) expression associated with human prostate cancer progression. *Oncology Research*.

[25] Koshiba T, Hosotani R, Wada M (1998). Involvement of matrix metalloproteinase-2 activity in invasion and metastasis of pancreatic carcinoma. *Cancer*.

[26] Cottam DW, Rennie IG, Woods K, Parsons MA, Bunning RAD, Rees RC (1992). Gelatinolytic metalloproteinase secretion patterns in ocular melanoma. *Investigative Ophthalmology and Visual Science*.

[27] Väisänen A, Kallioinen M, von Dickhoff K (1999). Matrix metalloproteinase-2 (MMP-2) immunoreactive protein—a new prognostic marker in uveal melanoma?. *The Journal of Pathology*.

[28] Sen T, Moulik S, Dutta A (2009). Multifunctional effect of epigallocatechin-3-gallate (EGCG) in downregulation of gelatinase—a (MMP-2) in human breast cancer cell line MCF-7. *Life Sciences*.

[29] Ho Y, Yang S, Peng C, Chou M, Chang Y (2007). Epigallocatechin-3-gallate inhibits the invasion of human oral cancer cells and decreases the productions of matrix metalloproteinases and urokinase-plasminogen activator. *Journal of Oral Pathology and Medicine*.

[30] Deng Y, Lin J (2011). EGCG inhibits the invasion of highly invasive CL1-5 lung cancer cells through suppressing MMP-2 expression via JNK signaling and induces G2/M arrest. *Journal of Agricultural and Food Chemistry*.

[31] Hu D (2000). Regulation of growth and melanogenesis of uveal melanocytes. *Pigment Cell Research*.

[32] Hu DN, Roberts JE (1997). Melatonin inhibits growth of cultured human uveal melanoma cells. *Melanoma Research*.

[33] Hu DN, McCormick SA, Roberts JE (1998). Effects of melatonin, its precursors and derivatives on the growth of cultured human uveal melanoma cells. *Melanoma Research*.

[34] Hu D, Wakamatsu K, Ito S, McCormick SA (2009). Comparison of eumelanin and pheomelanin content between cultured uveal melanoma cells and normal uveal melanocytes. *Melanoma Research*.

[35] Chen X, Wang J, Shen H (2011). Epigenetics, microRNAs, and carcinogenesis: functional role of microRNA-137 in uveal melanoma. *Investigative Ophthalmology and Visual Science*.

[36] Yan D, Zhou X, Chen X (2009). MicroRNA-34a inhibits uveal melanoma cell proliferation and migration through downregulation of c-Met. *Investigative Ophthalmology and Visual Science*.

[37] Ye M, Hu D, Tu L (2008). Involvement of PI3K/Akt signaling pathway in hepatocyte growth factor-induced migration of uveal melanoma cells. *Investigative Ophthalmology & Visual Science*.

[38] Cui Z, Song E, Hu D, Chen M, Rosen R, McCormick SA (2012). Butein induces apoptosis in human uveal melanoma cells through mitochondrial apoptosis pathway. *Current Eye Research*.

[39] Chao S-C, Huang S-C, Hu D-N, Lin H-Y (2013). Subtoxic levels of apigenin inhibit expression and secretion of VEGF by uveal melanoma cells via suppression of ERK1/2 and PI3K/Akt pathways. *Evidence-Based Complementary Alternative Medicine*.

[40] Takahashi A, Watanabe T, Mondal A (2014). Mechanism-based inhibition of cancer metastasis with (-)-epigallocatechin gallate. *Biochemical and Biophysical Research Communications*.

[41] Wu Y, Lin Y, Liu H, Li J (2008). Inhibition of invasion and up-regulation of E-cadherin expression in human malignant melanoma cell line A375 by (-)-epigallocatechin-3-gallate. *Journal of Huazhong University of Science and Technology—Medical Science*.

[42] Singh T, Katiyar SK (2011). Green tea catechins reduce invasive potential of human melanoma cells by targeting COX-2, PGE 2 receptors and epithelial-to-mesenchymal transition. *PLoS ONE*.

[43] Yu G, Hu D, McCormick SA (2006). Latitude and incidence of ocular melanoma. *Photochemistry and Photobiology*.

[44] Bergman L, Seregard S, Nilsson B, Ringborg U, Lundell G, Ragnarsson-Olding B (2002). Incidence of uveal melanoma in Sweden from 1960 to 1998. *Investigative Ophthalmology & Visual Science*.

[45] Van Raamsdonk CD, Griewank KG, Crosby MB (2010). Mutations in GNA11 in uveal melanoma. *The New England Journal of Medicine*.

[46] Vajdic CM, Hutchins A, Kricker A (2003). Chromosomal gains and losses in ocular melanoma detected by comparative genomic hybridization in an Australian population-based study. *Cancer Genetics and Cytogenetics*.

[47] Lin CW, Chen PN, Chen MK (2013). Kaempferol reduces matrix metalloproteinase-2 expression by down-regulating ERK1/2 and the activator protein-1 signaling pathways in oral cancer cells. *PLoS ONE*.

[48] Chen PN, Hsieh YS, Chiang CL, Chiou H, Yang S, Chu S (2006). Silibinin inhibits invasion of oral cancer cells by suppressing the MAPK pathway. *Journal of Dental Research*.

